# Pituitary P62 deficiency leads to female infertility by impairing luteinizing hormone production

**DOI:** 10.1038/s12276-021-00661-4

**Published:** 2021-08-27

**Authors:** Xing Li, Ling Zhou, Guiliang Peng, Mingyu Liao, Linlin Zhang, Hua Hu, Ling Long, Xuefeng Tang, Hua Qu, Jiaqing Shao, Hongting Zheng, Min Long

**Affiliations:** 1 Department of Endocrinology, Translational Research Key Laboratory for Diabetes, Xinqiao Hospital, Army Medical University, Xinqiao Main Street No. 183, Shapingba, Chongqing, China; 2 Department of Endocrinology, Jinling Hospital, Medical School of Nanjing University, Zhongshan East Street No. 305, Xuanwu, Nanjing, China; 3 Department of Endocrinology, Jinling Hospital, Nanjing Medical University, Zhongshan East Street No. 305, Xuanwu, Nanjing, China; 4 Department of Gynaecology and Obstetrics, Xinqiao Hospital, Army Medical University, Xinqiao Main Street No. 183, Shapingba, Chongqing, China; 5 Department of Pathology, Xinqiao Hospital, Army Medical University, Xinqiao Main Street No. 183, Shapingba, Chongqing, China

**Keywords:** Metabolic diseases, Pituitary gland

## Abstract

P62 is a protein adaptor for various metabolic processes. Mice that lack p62 develop adult-onset obesity. However, investigations on p62 in reproductive dysfunction are rare. In the present study, we explored the effect of p62 on the reproductive system. P62 deficiency-induced reproductive dysfunction occurred at a young age (8 week old). Young systemic p62 knockout (p62^-/-^) and pituitary-specific p62 knockout (p62^flox/flox^ αGSU^cre^) mice both presented a normal metabolic state, whereas they displayed infertility phenotypes (attenuated breeding success rates, impaired folliculogenesis and ovulation, etc.) with decreased luteinizing hormone (LH) expression and production. Consistently, in an infertility model of polycystic ovary syndrome (PCOS), pituitary p62 mRNA was positively correlated with LH levels. Mechanistically, p62^-/-^ pituitary RNA sequencing showed a significant downregulation of the mitochondrial oxidative phosphorylation (OXPHOS) pathway. In vitro experiments using the pituitary gonadotroph cell line LβT2 and siRNA/shRNA/plasmid confirmed that p62 modulated LH synthesis and secretion via mitochondrial OXPHOS function, especially Ndufa2, a component molecule of mitochondrial complex I, as verified by Seahorse and rescue tests. After screening OXPHOS markers, Ndufa2 was found to positively regulate LH production in LβT2 cells. Furthermore, the gonadotropin-releasing hormone (GnRH)-stimulating test in p62^flox/flox^ αGSU^cre^ mice and LβT2 cells illustrated that p62 is a modulator of the GnRH-LH axis, which is dependent on intracellular calcium and ATP. These findings demonstrated that p62 deficiency in the pituitary impaired LH production via mitochondrial OXPHOS signaling and led to female infertility, thus providing the GnRH-p62-OXPHOS(Ndufa2)-Ca^2+^/ATP-LH pathway in gonadotropic cells as a new theoretical basis for investigating female reproductive dysfunction.

## Reproductive biology: Linking metabolism and ovulation

A protein that helps maintain normal metabolic function also regulates the production of hormones that govern female fertility. Problems with ovulation are the most common cause of infertility, and these are in turn commonly associated with endocrine abnormalities and obesity. Researchers led by Min Long and Hongting Zheng of the Army Medical University in Chongqing, China, have now determined that a protein called P62 links these conditions. P62 is associated with obesity and diabetes, and the researchers showed that mice lacking this protein become overweight late in life but also exhibited impaired ovulation at an early age. Closer analysis revealed that P62 activity in the pituitary gland helps coordinate production of luteinizing hormone, which regulates ova maturation and release. These findings thus offer new insights into the connection between metabolic and reproductive health.

## Introduction

Infertility is a disease of the reproductive system. A World Health Organization (WHO) survey performed in developed countries estimates that female infertility accounts for 37% of causes in infertile couples, conveying that infertility is a serious problem affecting women’s quality of life^1–3^. Ovulatory disorders are identified as the most common factors that account for female infertility^4^. Importantly, the hypothalamic-pituitary-ovarian (HPO) axis tightly controls female reproduction, the dysfunction of which leads to ovulation disorders classified into three categories^5^, according to the WHO. Group I ovulation disorders encompass hypogonadotropic hypogonadism due to impaired secretion of pituitary gonadotropins. The genetic pathogeny of gonadotropin deficiency comprises defects in neuropeptides/proteins and genes controlling GnRH and/or participating in the synthesis of LH or FSH subunits^6,7^. Group II, which accounts for the majority of ovulation disorders at 85% of cases, is caused by polycystic ovarian syndrome (PCOS), overweight/obesity and endocrinopathies. Correspondingly, accumulating evidence suggests that metabolic disorders participate in reproductive dysfunction^8,9^. Currently, crosstalk among metabolism and the reproductive system is gradually being explored. For example, specific knockout of the insulin receptor in pituitary/ovarian theca cells could rescue obesity-induced female infertility^8,10^. However, ablating insulin receptors specifically in astrocytes leads to delayed puberty and hypogonadism^11^. These studies indicated the complex mechanisms in the reproductive system. Classical metabolic signals that modulate reproductive functions are intriguing and worth illuminating.

P62, as we previously reviewed, is a multifunctional metabolic adaptor that contributes to the pathogenesis of diverse metabolic disorders, including obesity, type 2 diabetes mellitus (T2DM), metabolic bone diseases, cancer, and neurodegenerative diseases^12–15^. Tissue-specific knockout mice with deletion of p62 in adipose tissue or the brain illustrate the important role of p62 in adipogenesis, adipose tissue transformation and central appetite regulation^12–14^. Interestingly, young female p62^−/−^ mice are nonobese with normal fat content and metabolic parameters but develop obesity in adulthood^12^. These results uncover a novel role for p62 in the control of metabolic pathways. However, how p62 functions in the reproductive system remains largely unknown. In our preliminary studies, a decreased accumulative number of pups with abnormal ovarian histomorphology was observed in adult female p62^−/−^ mice, suggesting a phenotype of reproductive dysfunction.

In the present study, p62 deficiency led to female infertility at a young age through damaged luteinizing hormone (LH) synthesis and secretion in pituitary gonadotropin cells. P62 modulates LH via the mitochondrial oxidative phosphorylation (OXPHOS) signaling pathway, especially its complex I component Ndufa2, along with subsequent oxygen consumption. P62 also acts as a modulator of the GnRH-LH axis, which is dependent on intracellular calcium and ATP. Our data demonstrate that p62 positively modulates pituitary LH in the regulation of female reproduction.

## Materials and methods

### Animal husbandry and ethics statement

All mice were housed in a pathogen-free animal facility at the Laboratory Animal Centre at Xinqiao Hospital. The animals were maintained in a temperature- and humidity-controlled room on a 12 h light/12 h dark cycle and fed a standard laboratory chow diet. All experiments involving animals were conducted according to the ethical policies and procedures approved by the Laboratory Animal Welfare and Ethics Committee of the Army Medical University, license number: SYXK (military) 2017-0062.

### Generation and genotyping of systemic and pituitary-specific p62^−/−^ mice

P62^−/−^ mice and αGSU^cre^ mice were purchased from Jackson Laboratories (Sacramento, CA USA). The generation of p62^−/−^ mice was described previously^15^. Genotyping was performed by PCR using tail-biopsy DNA. Genomic DNA was isolated from the tail using the Easy-DNA Kit (Sigma, Waltham, MA, USA). Pituitary-specific p62^−/−^ mice (p62^flox/flox^ αGSU^cre^) and control mice (p62^flox/flox^) were on a C57BL/6JGpt background created using the cre/loxP approach^16–18^. The genotyping banding pattern is shown in Supplementary Fig. 1a, b and Fig. 3a. The genotyping primers for systemic and pituitary-specific p62^−/−^ mice are listed in Supplementary Table 3.

### Representative breeding experiment

**Fig. 1:**
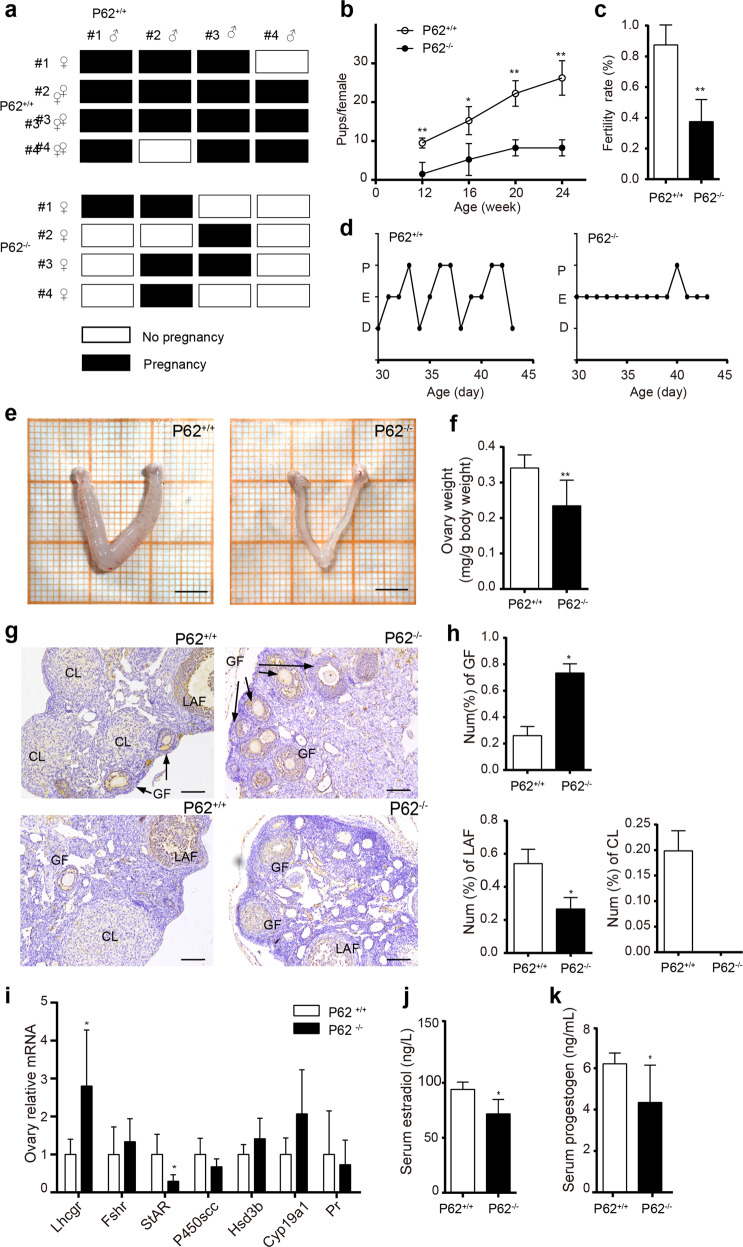
P62 deficiency leads to female infertility. **a** Representative breeding study illustration: four young (8 week old) p62^−/−^ or p62^+/+^ female mice were paired with four p62^+/+^ adult male mice for 7 days and then returned to their own cages for 3 weeks to allow for the birth of pups, indicating successful pairing. Each row represents one individual female, and each bar represents one of her pairings. Four examples were chosen from each group to illustrate the fertility phenotypes. **b** Cumulative number of pups per female and **c** infertility success rate of female mice in each group during the breeding study, *n* = 4. **d–f** Estrous cycles, representative images of ovaries with uteri (scale bar, 500 μm), and the relative quantification of ovary weights in each group. **g** H&E staining of mouse ovaries. CL corpus luteum, LAF large antral follicle, GF growing follicle; scale bar, 20 μm. **h** The percentage of ovarian growing follicles, large antral follicles, and corpus luteum counts among all the follicles in each group, *n* = 4–5. **i** The relative mRNA expression levels of key steroidogenesis signaling pathway proteins in the ovaries of 8-week-old p62^−/−^ and p62^+/+^ female mice, *n* = 4–6. **j**, **k** Serum estradiol (E2) and progestogen (P) levels of the young p62^−/−^ and p62^+/+^ female groups, *n* = 5–8. The data in **i** are presented as the mean ± SEM, and the data in the other panels are presented as the mean ± SD. Student’s *t-*test. *P < 0.05; **P < 0.01.

Four female mice were chosen from each group to illustrate the fertility phenotypes. Four young (8 week old) p62^−/−^ or p62^+/+^ female mice were paired with four age-matched p62^+/+^ male mice for 7 days and then returned to their own cages for 3 weeks to allow for the birth of pups, indicating successful pairing. P62^flox/flox^ αGSU^cre^ and p62^flox/flox^ mice were maintained in the same way. In Fig. 1a and Supplementary Fig. 5, each row represents one individual female, and each bar represents one of her pairings.

### Assessment of estrous cycles

The stage of the estrous cycle was determined by vaginal cytology. Normal mice showed consecutive regular 4-day estrous cycles. The complete estrous cycle was divided into four consecutive stages, namely, proestrus (P), estrus (E), metestrus (M), and diestrus (D), according to vaginal cytology: small, round, nucleated epithelial cells were mainly in proestrus (P); anucleated keratinized epithelial cells were mainly in estrus (E); metestrus (M) was characterized by a combination of anucleated keratinized epithelial cells and neutrophils; and diestrus (D) was characterized by fewer anucleated keratinized epithelial cells with a combination of neutrophils and small and large nucleated epithelial cells^19,20^. For 14 consecutive days from 9 to 10 a.m., vaginal cytology was assessed and staged; vaginal smears were collected with fine cotton swabs soaked in saline, coated on glass slides, fixed and H&E stained. After the experiments, the mice were all sacrificed at E stage.

### Assessment of ovarian histology

Ovarian histological assessment was performed by H&E staining of 5 μm-thick tissue sections. The corpus luteum (CL) formed immediately after ovulation from the ruptured Graafian follicle. Follicles of different stages were counted and classified based on the mean diameters. In systemic and pituitary-specific p62^−/−^ and p62^+/+^ mice, follicles were categorized into two distinct classes: growing follicles (GFs, 20–310 μm) and large antral follicles (LAFs, 310 μm)^21–24^. In PCOS mice, follicular cysts (FCs) were additionally identified, which were also called cystic follicles, a pathologic follicle structure that showed an expanding diameter with degrading granulosa cell layers^25^.

### PCOS experimental animal model

PCOS was induced in 22-day-old mice by injecting dehydroepiandrosterone (DHEA, Sigma, Ronkonkoma, New York, USA) (6 mg/100 g body weight, dissolved in 0.01 ml 95% ethanol, which was further diluted with corn oil) subcutaneously for 20 consecutive days^26,27^. The DHEA-PCOS murine model presents several features of human PCOS, such as hyperandrogenism, abnormal maturation of ovarian follicles and anovulation^25–27^. We administered corn oil with 95% ethanol to the control (WT + placebo) group. At the end of the experiments, vaginal smears and hematoxylin and eosin (H&E) staining were conducted every day for 2 weeks. The animals were sacrificed at the estrus stage by cervical dislocation followed by organ excision and serum collection, which was frozen at −80 °C.

### RT-PCR and western blotting

Tissues were homogenized in TRIzol reagent (TIANGEN, Beijing, China), and total RNA was extracted according to the manufacturer’s instructions. The RNA quality was assessed with a NanoDrop 2000 spectrophotometer (Thermo Fisher Scientific, Waltham, MA, USA) by examining the 260-to-280 ratio. Samples with a ratio of 1.8–2.0 were reverse-transcribed using a Prime Script RT reagent Kit (Takara, Terra Bella Ave, CA, USA). The target genes were analyzed by RT-PCR using SYBR Premix Ex Taq II (Takara, Terra Bella Ave, CA, USA) in an Applied Biosystems 7300 system (Thermo Fisher Scientific, Waltham, MA, USA). The primers are listed in Supplementary Table 4, and GAPDH mRNA expression served as the normalization reference. WB was performed as previously described^28^. Briefly, cells were harvested in sample buffer (2% SDS, 50 mmol/L Tris-HCl [pH 6.8], 100 mmol/L dithiothreitol, 10% glycerol, and 0.1% bromophenol blue), boiled at 98 °C for 5 min, and sonicated for 2 min at 10% intensity, and protein concentrations were determined by an RC DC Protein Assay kit (Bio-Rad, Hercules, CA, USA). Cell lysates underwent SDS-PAGE and WB with the indicated primary antibodies (1:800–1:8000) and their corresponding secondary antibodies. Signal acquisition was performed with chemiluminescent HRP substrate (Millipore, Waltham, MA, USA), and the results were imaged with an FX5s system (Vilber Lourmat, Manchester, UK). Antibodies recognizing mouse LH (catalog LH-112AP) and FSH (catalog FSH-101AP) were obtained from Fabgennix (Frisco, TX, USA). β-Actin (catalog 4970) was purchased from Abcam (Abcam, Cambridge, MA, USA) and Cell Signaling Technology (Danvers, MA, USA).

### Immunofluorescence

Mouse pituitary tissues were embedded in OCT, quickly frozen, and cut into 3.5 mm-thick sections. Cell slides were fixed with 4% paraformaldehyde at room temperature for 15 min. Tissue sections were permeabilized with 0.2% Triton-X 100 for 5 min, and the cell slides were incubated in ice-cold methanol for 2 min. After PBS washing, these specimens were incubated with 5% BSA for 30 min at room temperature, incubated with the indicated primary antibodies at 4 °C overnight, washed with PBS, and then incubated with secondary antibodies at room temperature for 1 h. Subsequently, DAPI (Beyotime, Shanghai, China) was applied for 5 min at room temperature. Finally, the specimens were mounted on coverslips with Fluoromount (Boster, Pleasanton, California, USA), and fluorescence images were obtained using a TC5-SP5 confocal laser scanning microscope (Leica Microsystems, Wetzlar, Germany).

### RNA sequencing and data analysis

Total RNA of cells was harvested with TRIzol (Takara, Terra Bella Ave, CA, USA) and quantified by a NanoDrop 2000. RNA integrity and gDNA contamination were tested by denaturing agarose gel electrophoresis (Thermo Fisher Scientific, Waltham, MA, USA), and the sequencing library was determined by an Agilent 2100 Bioanalyzer using an Agilent DNA 1000 chip kit (Agilent, Santa Clara, CA, USA). RNA-seq analyses were performed on HiSeq 4000 platforms (Illumina, San Diego, CA, USA). Kyoto Encyclopedia of Genes and Genomes (KEGG) pathway analysis is a functional analysis mapping genes to KEGG pathways and was performed by DAVID^29^. Heatmap was built using pheatmap in the R library.

### Glucose tolerance test (GTT) and insulin tolerance test (ITT)

Mice that had been fasted overnight were injected with either 2 g/kg body weight glucose or 0.75 U/kg body weight of human regular insulin into the peritoneal cavity. Glucose levels were determined in blood collected from the tail tip immediately before and 15, 30, 60, and 120 min after the injection using glucose testing strips (ACCU-CHEK, Roche, Shanghai, China).

### Hormone and metabolic indicator measurements

All blood samples were collected at 9–10 a.m^30^. during the estrus period. Serum testosterone, estradiol, progesterone, LH, FSH, TSH, PRL, and ACTH concentrations were quantified using ELISA kits from Nanjing Jiancheng Biotechnology Company (Shanghai, China) following the manufacturer’s instructions. Fasting blood glucose was measured using glucose testing strips (ACCU-CHEK, Roche, Shanghai, China). Serum TG was measured by a TG kit (Nanjing Jiancheng, Shanghai, China).

### Cell culture and transfection

The pituitary gonadotroph cell line LβT2 was provided by P. Mellon (UCD, Davis, California, USA)^31^ and cultured in DMEM with 10% fetal bovine serum (Gibco, Waltham, MA, USA). Before cell passage, the bottom of the culture plate was coated with L-lysine (R&D, Minneapolis, Canada). Cells were maintained at 37 °C in a humidified atmosphere under 5% CO2. LβT2 cells seeded at a density of 7.0 × 10^5^ cells on a 12-well plate were electroporated with 120 pmol of siRNA using the RNAiMAX and Lipofectamine 3000 Transfection System (Thermo Fisher Scientific, Waltham, MA, USA). Oligonucleotide siRNA duplexes and plasmids were synthesized by Shanghai Shenggong Technology. Mouse p62 short hairpin RNA (shRNA) and control shRNA (GenePharma, Shanghai, China) were constructed. Cells were used at 40% confluency, and 2 µl of shRNA was added to each well in a 96-well plate, followed by incubation for 6 h. After transfection, LβT2 cells and the supernatant were collected for use. The sequences are listed in Supplementary Table 5.

### NAD^+^/NADH measurements

The NAD^+^ and NADH contents were measured using an NAD^+^ /NADH Assay Kit (Beyotime, Shanghai, China) according to the manufacturer’s instructions.

### Cell oxygen consumption rate (OCR) measurement

The cellular oxidation state was measured using a Seahorse XF96 extracellular flux analyzer (Seahorse Biosciences, North Billerica, Massachusetts, USA) according to the manufacturer’s protocol. Briefly, 10,000 cells were seeded in each well of XF 24-well microplates. The final concentrations of the mitochondrial inhibitors were 1 μM oligomycin, 3 μM FCCP, and 0.5 μM rotenone. Basal respiration indicates the baseline oxygen consumption reading prior to compound injection. Maximal respiration represents the maximum OCR measurement value after FCCP injection. After detection, the cell protein concentrations were assessed, and the OCR was adjusted accordingly. ATP production was represented by the change in the values between the maximal and baseline OCRs.

### GnRH-stimulating experiment and calcium/ATP block test

Gonadorelin is a synthetic peptide with a structure that is similar to natural GnRH in various mammalian species. In the in vivo experiment, during the estrus period in the morning, mice were muscularly injected with gonadorelin (BBCA pharmaceutical company, Anhui, China) at a dose of 7.5 μg/kg body weight. Blood samples were collected from the tail vein before injection and 40 min after injection, according to the manufacturer’s instructions for gonadorelin and previous studies^32,33^. In an in vitro experiment, we utilized gonadorelin (100 nM) to treat LβT2 cells for 48 h and then collected culture supernatant and cell lysates. The calcium chelator BAPTA (2 mM for 1 h, Sigma, Ronkonkoma, New York, USA) or the mitochondrial NADH:ubiquinone reductase inhibitor rotenone (5 μM for 2 h, Sigma, Ronkonkoma, New York, USA) inhibited ATP synthesis, and then the supernatant and cell lysates were collected for further experiments.

### Data presentation and analysis

Values are shown as the mean ± SD or the mean ± standard error of the mean (SEM), as appropriate. To determine statistical significance, the statistical tests included Student’s *t*-test, one-way analysis of variance (ANOVA), and Pearson analysis. A *P* value less than 0.05 was considered statistically significant. Different letters above the columns indicate significance in the group difference at *P ≤ 0.05; **P ≤ 0.01; or ***P < 0.001. Statistical analysis was performed using GraphPad Prism 7.

## Results

### P62 deficiency leads to primary reproductive dysfunction at a young age

Genotyping verified that p62 knockout female mice (Supplementary Fig. 1a, b) showed significantly elevated body weight at 22 weeks, which increased by 21.77% at 32 weeks with an obviously obese appearance (Supplementary Fig. 1c, d). Signs of reproductive dysfunction in adult (32 week old) p62^−/−^ female mice were observed, such as a decreased number of pups (Supplementary Fig. 1e) and a lack of the corpus luteum in the ovaries (Supplementary Fig. 1f-h). Considering that the p62^−/−^ female mice began to develop an abnormal metabolic phenotype after 22 weeks but were sexually mature at 8 weeks old, to determine whether reproductive dysfunction was directly related to the lack of p62 or was secondary to the potential obese phenotype of the mutant mice, we investigated the reproductive function of young p62^−/−^ female mice before obvious metabolic disorders occurred, referring to a previous study^12^. Via a representative breeding experiment, interestingly, we found that 8-week-old p62^−/−^ female mice already displayed infertility characteristics, including attenuated breeding success rates and cumulative number of pups per female (Fig. 1a-c), although they had a normal body weight, triglyceride levels, blood glucose levels (Supplementary Fig. 2a-c) and glucose/insulin tolerance (Supplementary Fig. 2d, e). These results indicated that p62 deficiency might lead to female reproductive dysfunction, which was not secondary to obesity. Furthermore, 8-week-old p62^−/−^ female mice showed an abnormal estrous cycle remaining in the estrus period compared to the normal cycle of p62^+/+^ mice (Fig. 1d). Relevantly, a reduced size of estrogen hormone target organs was exhibited, as evidenced by threadlike atrophied uteri and reduced ovary weights (Fig. 1e, f). Histological analysis revealed that the ovaries of p62^−/−^ mice formed more growing follicles (GFs) than those of p62^+/+^ mice (Fig. 1g, h). However, p62^−/−^ mice lacked late-stage antral follicle (LAF) or corpora luteum (CL) formation (Fig. 1h), which was consistent with the failure of folliculogenesis and ovulation. Additionally, by detecting steroidogenic genes (Lhcgr, Fshr, StAR, P450scc, Hsd3b, Hsd17b, Cyp19a1, and Pr) in ovaries, decreased StAR, which encodes steroidogenic acute regulatory protein (StAR), and increased Lhcgr (LH receptor) expression were found in p62^−/−^ ovaries (Fig. 1i). An abnormal steroidogenic pathway was accompanied by lower serum estradiol and progestogen levels (Fig. 1j, k). These data suggested that p62 deficiency might lead to female infertility at a young age.

### P62 deficiency impairs pituitary LH production in female mice

**Fig. 2:**
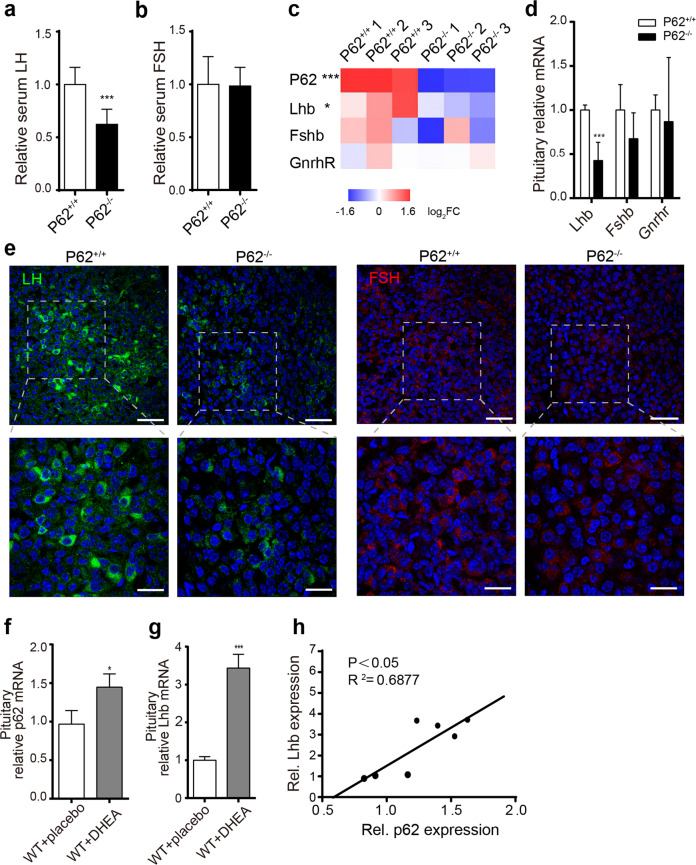
Abnormal p62 expression dysregulated LH production in the pituitary of female mice. **a**, **b** Serum LH and FSH levels of young (8 week old) p62^−/−^ and p62^+/+^ female mice, *n* = 6–9. **c**, **d** RNA sequencing and RT-PCR detection of the relative expression of gonadotropin markers (Lhb, Fshb, and Gnrhr) in the pituitary. **e** Representative immunohistological images of pituitary sections stained for LH (green) and FSH (red) of young p62^+/+^ or p62^−/−^ female mice. DAPI (blue), scale bars: 40 μm (left); 20 μm (right). **f**, **g** Relative mRNA expression of p62 and Lhb in the pituitary of PCOS model mice (WT + DHEA) and controls (WT + placebo), *n* = 3–4. **h** The correlation between pituitary p62 and relative Lhb mRNA expression in PCOS mice and control mice, *n* = 7. Statistical analyses were performed using Student’s *t-*test (**a**, **b**, **d**, **f**, **j**) and Pearson analysis (**h**). Data are presented as the mean ± SD. *P < 0.05; ***P < 0.001.

To evaluate the cause of infertility, we measured serum LH by ELISA and detected significantly reduced serum LH levels in young p62^−/−^ female mice (Fig. 2a), while FSH stayed nearly unchanged (Fig. 2b). Serum thyroid stimulating hormone (TSH), prolactin (PRL) and adrenocorticotropic hormone (ACTH) also showed no significant differences (Supplementary Fig. 2f-h). By RNA sequencing (RNA-Seq) of mouse pituitary tissues, p62 mRNA robustly decreased in p62^−/−^ pituitary tissues, accompanied by decreased luteinizing subunit beta (Lhb) mRNA expression (Fig. 2c), which was confirmed by RT-PCR (Fig. 2d). Immunofluorescence staining showed reduced numbers of LH-expressing cells in pituitary tissues of p62^−/−^ mice (Fig. 2e). Additionally, in an infertile DHEA-PCOS model, elevated LH and testosterone with decreased estrogen (Supplementary Fig. 3a-d) led to a disordered estrous cycle lacking the metestrus and diestrus periods (Supplementary Fig. 3e, f), as well as abnormally larger follicles with rare corpora luteum in ovaries (Supplementary Fig. 3g). Increased Lhb mRNA levels were accompanied by p62 mRNA elevation in the pituitaries of PCOS mice (WT + DHEA) (Fig. 2f, g); intriguingly, a significantly positive correlation between p62 and LH was found (Fig. 2h). To verify the cause of low LH in p62^−/−^ mice, we also analyzed the LH-related regulatory genes (GnRH, Kiss1, Gpr54, Tac2, Tacr3, Pdyn, and Kor) in the hypothalamus and found that only Kiss1 mRNA was significantly increased in p62^−/−^ mice (Supplementary Fig. 4a). Elevated Kiss1 in young p62^−/−^ female mice might be a compensatory response toward reduced circulating LH, as Kiss1 generally modulates LH secretion^34^. Moreover, RNA-Seq of p62^−/−^ pituitaries showed no significant change in the genes related to pituitary development and early pituitary function (Supplementary Fig. 4b, c, Supplementary Table 1). These results indicated that p62 regulated pituitary gonadotropic LH production, which might be the dominant aspect influencing female reproductive function.

**Fig. 3:**
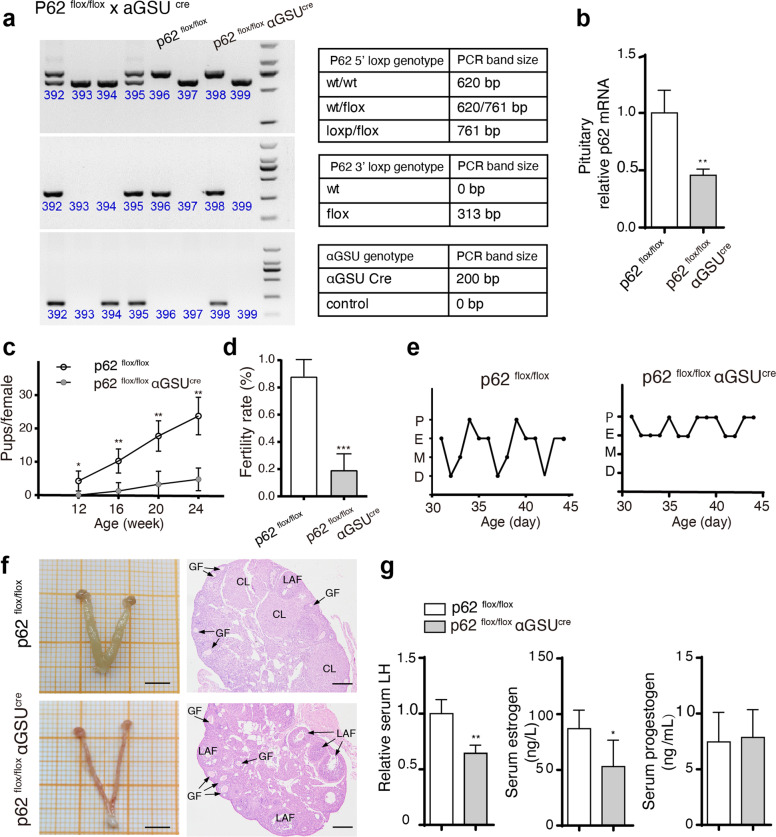
Reproductive dysfunction is exhibited in pituitary-specific p62 knockout mice. Pituitary-specific p62^−/−^ mice (p62^flox/flox^ αGSU^cre^) and controls (p62^flox/flox^) were created using the cre/loxP approach. **a** Genotyping and PCR bands of p62^flox/flox^ and p62^flox/flox^ αGSU^cre^ mice. **b** Relative p62 mRNA expression in the pituitary of young female p62^flox/flox^ and p62^flox/flox^ αGSU^cre^ mice, *n* = 4. **c**, **d** Infertility ability was analyzed by counting cumulative pups per female and the successful fertility rate. **e** The estrous cycle of young female mice, *n* = 4. **f** Representative morphology images of the ovaries and uterus by H&E staining of the ovaries in young female p62^flox/flox^ and p62^flox/flox^ αGSU^cre^ mice. GF growing follicle, LAF large antral follicle, CL corpus luteum; Scale bar: 500 μm (left), 200 μm (right). **g** Serum LH, E2, and P levels in each group, *n* = 4–6. Statistical analyses were performed using Student’s *t-*test. Data are shown as the mean ± SD. Student’s *t-*test. *P ≤ 0.05; **P ≤ 0.01; ***P < 0.001.

To confirm the central role of p62 specifically in the pituitary gland, anterior pituitary-specific p62 knockout (p62^flox/flox^ αGSU^cre^) and control mice (p62^flox/flox^) were constructed. The reliability of the mouse model was verified (Fig. 3a, b). Breeding experiments displayed an attenuated breeding success rate and pup number in young p62^flox/flox^ αGSU^cre^ female mice (Supplementary Fig. 5, Fig. 3c, d). An incomplete estrous cycle in p62^flox/flox^ αGSU^cre^ mice repeated between the proestrus and estrus periods (Fig. 3e). Consistently, p62^flox/flox^ αGSU^cre^ mice had a thinner uterus, and the formation of follicles increased but showed less luteinizing corpus (Fig. 3f), with decreased serum LH and estrogen (Fig. 3g). In summary, p62^flox/flox^ αGSU^cre^ mice mimicked most infertility characteristics of systemic p62^−/−^ mice, confirming that p62 deficiency in the pituitary directly led to decreased LH production and consequent infertility.

### P62 regulates LH synthesis and release through the mitochondrial oxidative phosphorylation (OXPHOS) pathway

**Fig. 4:**
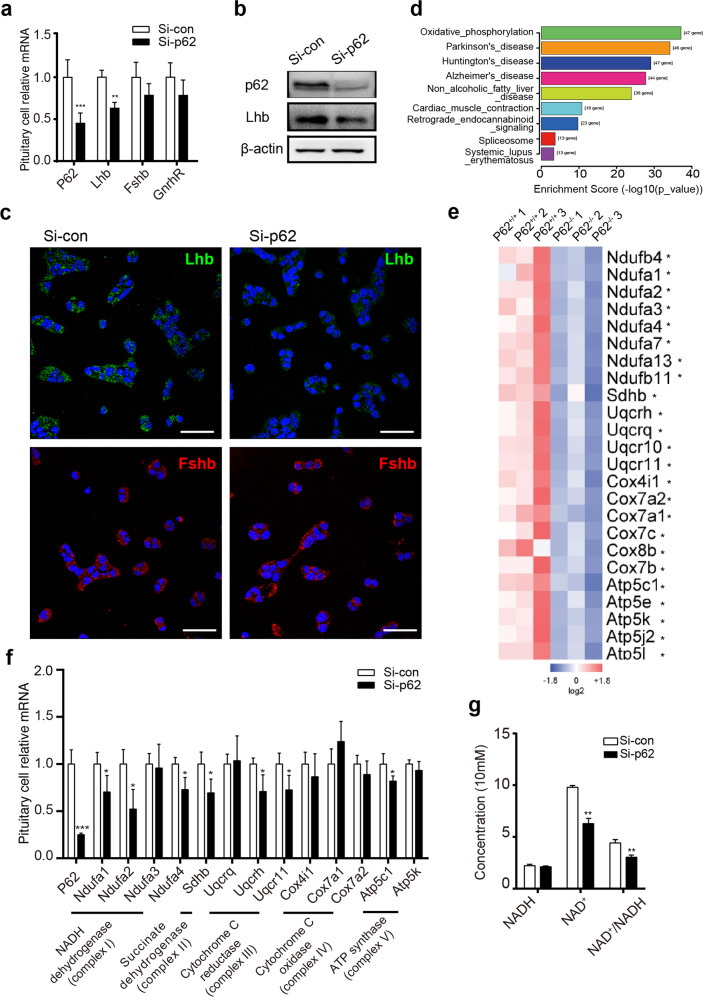
P62 deficiency leads to downregulated LH and oxidative phosphorylation (OXPHOS) in pituitary gonadotropin cells. **a–c** In vitro experiments in which the mouse pituitary gonadotroph LβT2 cell line was transfected with control/p62 siRNA for 48 h. **a** RT-PCR detection of p62, Lhb, Fshb, and GnRHR mRNA, *n* = 4–6. **b** WB detection of silenced p62 expression and LH protein levels. **c** Immunofluorescence detection of LH (green) and FSH (red) protein expression in LβT2 cells. DAPI in blue, scale bar: 20 μm. **d** The significant changed pathways in p62^−/−^ mouse pituitaries enriched by KEGG analysis. **e** Differentially expressed pituitary OXPHOS markers between p62^−/−^ and p62^+/+^ mice shown as a heatmap from RNA-Seq of young female mice, *n* = 3. **f** Relative mRNA expression levels of OXPHOS markers (mitochondrial complex I: Ndufa1–4; complex II: Sdhb; complex III: Uqcrq, Uqcrh, Uqcr11; complex IV: cox4i1, cox7a1, cox7a2; complex V: atp5c1, atp5k) with p62 siRNA treatment, *n* = 4. **g** NADH, NAD^+^ concentration and NAD^+^ /NADH ratio measurement, *n* = 4. All data are presented as the mean ± SD. Student’s *t-*test, *P < 0.05; **P < 0.01; ***P < 0.001.

To explore the mechanism of the p62-LH axis specifically in pituitary gonadotroph cells, we used the mouse pituitary gonadotroph cell line LβT2. After p62 siRNA treatment in LβT2 cells, RT-PCR showed decreased mRNA expression of p62 and Lhb (Fig. 4a). Relatively lower expression of Lhb protein was also detected via WB (Fig. 4b) and immunofluorescence staining (Fig. 4c). By pituitary tissue RNA-Seq analysis, the mitochondrial OXPHOS signaling pathway was identified and found to be significantly and distinctly downregulated in p62^−/−^ mouse pituitaries (Fig. 4d, e). The significantly decreased signaling molecules of the OXPHOS pathway include NADH dehydrogenase (Ndufb4, Ndufa1, Ndufa2, Ndufa3, etc.), succinate dehydrogenase (Sdhb), cytochrome C reductase (Uqcrh, Uqcr10, and Uqcr11), cytochrome C oxidase (Cox7a1, Cox7a2, Cox7c, Cox8b, etc.), and a combination of ATPases (Atp5c1, Atp5e, Atp5k, etc.) (Fig. 4e, Supplementary Table 2). To further confirm these changes in pituitary gonadotroph cells, we found that after p62 siRNA treatment of LβT2 cells for 48 h, the mRNA levels of Ndufa2, Sdhb, and Uqcrh were reduced by 47.8%, 30.74%, and 29.06%, respectively. Ndufa2, Sdhb, and Uqcrh were the most changed OXPHOS markers of mitochondrial complexes I, II, and III (Fig. 4f). Consistently, NADH remained stable while NAD^+^ production decreased, thus leading to a decreased NAD^+^ /NADH ratio in LβT2 cells after p62 siRNA treatment (Fig. 4g), suggesting that p62 loss damaged NADH dehydrogenase (mitochondrial complex I). Moreover, among Ndufa2, Sdhb, and Uqcrh siRNA transfection, only Ndufa2 siRNA treatment induced significantly decreased Lhb mRNA with lower supernatant LH levels, while Sdhb and Uqcrh did not influence Lhb significantly (Supplementary Fig. 6a, b). Additionally, the Ndufa2 overexpression plasmid led to increased Lhb mRNA (Supplementary Fig. 6c), exerting Ndufa2’s positive modulation on LH. To detect OXPHOS, Ndufa2 siRNA/plasmid treatment led to a correspondingly reduced/elevated oxygen consumption ratio (OCR) and ATP production (Supplementary Fig. 6d-k), as ATP contributed to the gonadotropin secretion process^35,36^. These data suggested that p62 regulated pituitary LH synthesis and release, possibly via the mitochondrial OXPHOS signaling pathway, and that Ndufa2 might be a vital molecule in the p62-LH axis.

### P62 modulates LH via Ndufa2 and participates in GnRH-induced LH synthesis and secretion

**Fig. 5:**
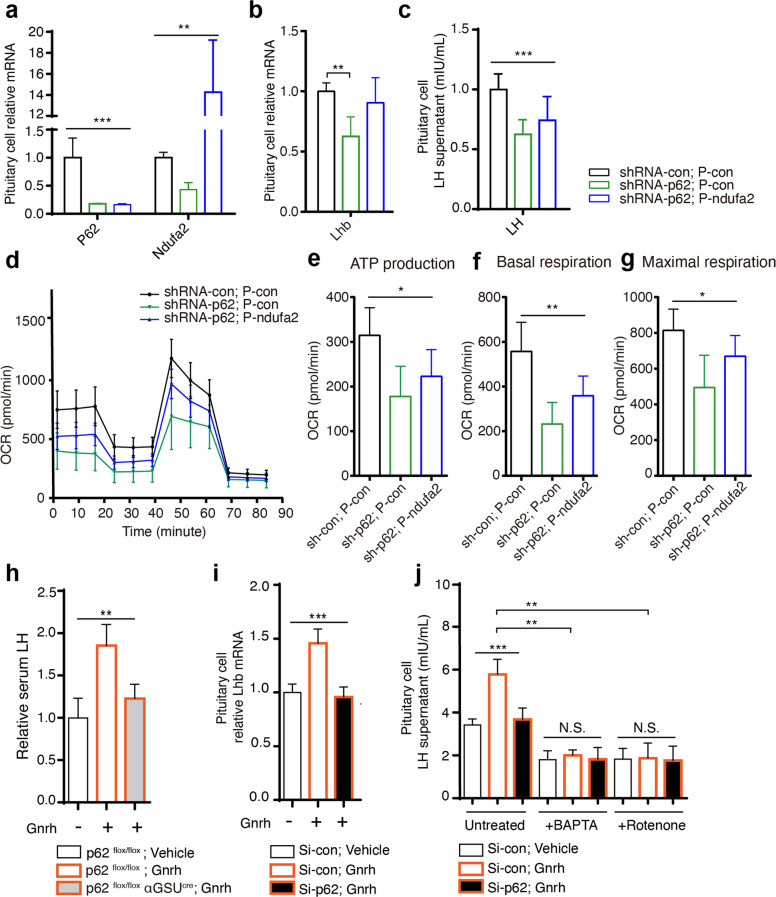
P62 targets the OXPHOS signaling pathway to regulate LH and GnRH-induced LH production. Rescue experiments were conducted in LβT2 cells by silencing p62 with shRNA, and p62 was stably silenced in cells overexpressing Ndufa2 with a plasmid for 48 h, thus generating three groups of LβT2 cells: shRNA-con, p-con; shRNA-p62, p-con; shRNA-p62, p-Ndufa2. **a** RT-PCR detection of p62 and Ndufa2 to confirm transfection efficiency, *n* = 3. **b**, **c** mRNA detection of Lhb (*n* = 3) and measurement of supernatant LH (*n* = 6–8) in each group. **d–g** Seahorse assay analysis of mitochondrial respiratory function in LβT2 cells, including ATP production, basal respiration, and maximal respiration, in each group, *n* = 5. **h** Relative serum LH in p62^flox/flox^ αGSU^cre^ and p62^flox/flox^ female mice treated with vehicle or the GnRH agonist gonadorelin (7.5 µg/kg body weight), n = 3-4. **i** Relative Lhb mRNA after gonadorelin induction in LβT2 cells, and **j** supernatant LH concentration level after treatment with the calcium chelator BAPTA (2 mM) or the mitochondrial NADH: ubiquinone reductase inhibitor rotenone (5 μM), with/without gonadorelin (100 nM), *n* = 4. All data are presented as the mean ± SD. Statistical analyses were performed using one-way ANOVA and Student’s *t-*test (**b**, **i**). *P < 0.05; **P < 0.01; ***P ≤ 0.001.

**Fig. 6:**
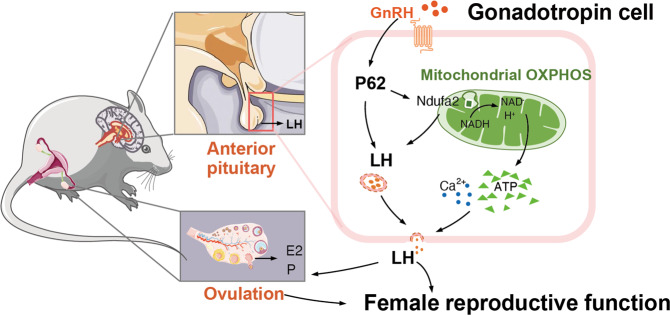
The modulative role of p62 in female reproductive biology. Schematic diagram of the GnRH-p62-OXPHOS (Ndufa2)-Ca^2+^/ATP-LH pathway in pituitary gonadotropic cells for regulating female reproduction.

Rescue experiments were conducted by shRNA knockout of p62 and plasmid overexpression of Ndufa2 in LβT2 cells. RT-PCR verified the transfection efficiency and confirmed the promoting effect of p62 on Ndufa2 mRNA expression (Fig. 5a). The attenuated LH expression and secretion induced by p62 silencing could be partly reversed by overexpressing Ndufa2 (Fig. 5b, c). Consistently, mitochondrial respiratory indicators, including OCR, ATP, and maximal respiration, were also reversed (Fig. 5d-g), indicating that p62 targeted the mitochondrial Ndufa2 signaling molecule to affect oxygen consumption and ATP generation, which might further regulate LH production. Finally, GnRH stimulation experiments using p62^flox/flox^ αGSU^cre^ and p62^flox/flox^ mice showed that pituitary p62 knockout significantly inhibited the GnRH-stimulated LH response in vivo (Fig. 5h). Using the LβT2 cell line, we also found that p62 silencing blocked GnRH-induced LH transcriptional elevation in vitro (Fig. 5i). Additionally, although p62 knockout efficiently inhibited GnRH-induced LH synthesis and secretion, blocking intracellular calcium by BAPTA or inhibiting ATP by rotenone could both greatly reduce LH secretion in LβT2 cells, regardless of the basal or GnRH-stimulated conditions with/without p62 (Fig. 5j), indicating that LH secretion was dependent on intracellular calcium and ATP. In summary, our data suggested the potential mechanism of the GnRH-p62-OXPHOS (Ndufa2)-Ca^2+^/ATP-LH pathway in pituitary gonadotropic cells that regulates female reproductive function, which is also illustrated as a schematic diagram (Fig. 6).

## Discussion

In recent decades, the prevalence of reproductive dysfunction has increased, accompanied by an increased occurrence of metabolic disorders worldwide^37,38^. A number of females suffer from reproductive dysfunction manifesting as infertility, which displays a complex pathogenesis and has aroused the attention of various researchers. Female infertility is characterized by ovulation disorders due to different pathological causes, including hypogonadotropic hypogonadism derived from hypothalamic-pituitary failure (HPF), for example, idiopathic hypogonadotropic hypogonadism (IHH), categorized as a group I ovulation disorder according to the WHO^5^; ovarian insufficiency (group III), defined as early depletion of ovarian function before the age of 40, associated with elevated FSH or low estradiol levels; and eugonadal ovulation disorder (group II), with the highest prevalence, which is characterized by almost healthily functioning gonads. PCOS is a common eugonadal ovulation disorder in reproductive-aged women with a prevalence of 6–10%^39^, and 60–70% of afflicted women have intrinsic obesity/insulin resistance^40^. Previous studies have identified vital signaling molecules/pathways participating in the crosstalk between metabolic and reproductive disorders. For example, GLUT4 is a protein required for insulin-mediated glucose translocation in adipocytes and contributes to insulin resistance in PCOS^41^. Insulin receptors synergetically act in different parts of the hypothalamus-pituitary-gonad (HPG) axis to regulate reproductive functions^8,10,11^. Autophagy, a major degradation/recycling pathway^42,43^, plays an essential role in cellular homeostasis and the reproductive system^44–46^. In the present study, we investigated p62, which is not only a multifunctional adaptor in metabolic diseases but is also a vital component of autophagy. Considering that p62 remains an unexplored field in reproduction, as well as our preliminary findings in adult p62^−/−^ female mice, we proposed that p62 might play a role in female infertility and mechanically link the connection between metabolic aspects and the reproductive system.

In this study, the pathogenesis of infertility in adult p62^−/−^ mice remained complex and might possibly be a syndrome mixed with genetic and obesity-induced metabolic factors. Since young nonobese p62^−/−^ mice have been confirmed to be in an almost normal metabolic state (normal weight, fat content, serum TG, fasting blood glucose, glucose/insulin tolerance, unchanged IR signaling pathways such as Akt and S6K activities in muscle and fat) in previous studies^12^ and in our Supplementary Fig. 2, we observed young p62^−/−^ female mice and surprisingly found that, even before the metabolic disorders arose, p62 deficiency can also lead to female infertility, including dysfunction of the pituitary (decreased LH levels at estrus stage) and ovary (abnormal menstrual cycle, folliculogenesis, anovulation, and steroidogenesis), suggesting primary infertility that is not secondary to obesity/metabolic disorders. Therefore, young nonobese p62^−/−^ mice are more similar to group I ovulation disorder-induced infertility caused by hypogonadism, whereas adult obese p62^−/−^ mice are more prone to PCOS (group II), which is due to metabolic disorders; thus, p62 might be an animal model for investigating the reproductive system, as well as the crosstalk between metabolism and reproduction. Importantly, using systemic/pituitary-specific p62 knockout mice and PCOS model mice, we found positive modulation of pituitary p62 on the synthesis and secretion of LH. Additionally, it could be concluded that inappropriate pituitary p62 levels lead to disordered gonadotropin with subsequent ovarian and hormone problems, indicating that balanced pituitary p62 levels might be critical in the regulation of LH and female peripheral gonadal functions.

Infertility could be a complex pathological state caused by various factors. We identified that the pituitary is the dominant organ/tissue that accounts for p62 deficiency-induced female infertility, while pathological changes still exist in the ovary and hypothalamus, which might be due to p62 loss or secondary phenomena. For example, in systemic p62 knockout mice, we observed that p62 deficiency-induced LH reduction was accompanied by an elevation of Lhcgr in the ovary and increased Kiss1 expression in the hypothalamus. The ovary is the target organ of LH to produce steroid hormones, triggered by the ligand-receptor activation of LH-Lhcgr^47^; thus, Lhcgr elevation might be a compensatory response, which is consistent with a previous study^19^. The lack of circulating LH primarily leads to depressed sex hormone production and the steroidogenesis signaling pathway in the ovary, such as decreased serum estrogen, progesterone and StAR mRNA expression in our results and ovulatory dysfunction. However, objectively, these changes might also be a direct result of p62 loss in the ovary. In the central nervous system, hypothalamic KNDy neurons control the GnRH pulse, and kisspeptin encoded by the kiss1 gene appears to directly activate GnRH neurons and subsequently the LH surge^48^. P62 loss rarely influenced KNDy neuron genes except for Kiss1, which could also be a compensatory response to decreased circulating LH. Since a similar infertility phenotype was observed in pituitary-specific p62 knockout mice, we specifically used a mouse gonadotropin cell line. Although there were quite a few factors that resulted in infertility, we could still confirm the role of the p62-LH axis in regulating pituitary gonadotropin. It is also worth further investigating the overall functions of p62 in the reproductive endocrine system.

Mechanistically, the OXPHOS signaling pathway greatly contributed to the modulatory effect of p62 on LH production. Among the various downregulated molecules in five mitochondrial components of OXPHOS, Ndufa2 from complex I changed distinctly. Additionally, p62 knockout led to a decrease in Ndufa2 with a lower NAD^+^/NADH ratio, representing an impaired NADH-to-NAD^+^ process catalyzed by mitochondrial NADH dehydrogenase/complex I, which is the first enzyme of the mitochondrial electron transport chain^49^. Ndufa2 positively regulated LH, oxygen consumption and ATP. Moreover, the p62-OXPHOS (Ndufa2)-LH pathway was identified via the rescue test. Importantly, using GnRH stimulation, we found that pituitary p62 acted as a necessary module in the GnRH-LH axis. Previous studies have identified that cellular ATP and calcium balance are critical for LH production, as mitochondrial ATP supplies energy for nuclear gene transcription and protein exocytosis^50,51^. Additionally, the interaction of GnRH with GnRHR in pituitary gonadotropic cells triggers a series of signal transductions and subsequent Ca^2+^ mobilization, which requires the energy resource ATP to control LH synthesis and exocytosis^52–55^. Our results were consistent with these notions, since LH production was dependent on ATP and calcium, regardless of GnRH stimulation or p62 expression. In conclusion, p62-OXPHOS (Ndufa2)-ATP/Ca^2+^-LH signaling might be a basic signaling pathway in pituitary gonadotropin cells.

Regretfully, although young nonobese p62^−/−^ mice were not obese and did not have distinct metabolic changes, we could not completely exclude the metabolic aspect, since the adipogenesis ERK signal has been subtlely stimulated in p62^-/-^adipocytes^12^. Even so, links between enhanced ERK in adipocytes and infertility have rarely been found, and the tissue-specific knockout experiments and the gonadotropic cell line in our study still helped to elucidate the functions of p62 in infertility^15^. In addition, due to the complexity of the pathogenesis of PCOS, the androgen-injected mouse model cannot completely mimic PCOS^56^. The mechanical investigation of this study was not in-depth enough, but it might have fully explained the gonadotropic part, which could still provide clues for future research in this field. However, clinical applications are limited, caution is warranted, and gene therapy precisely targeting pituitary cells still requires technical solutions. P62 might play comprehensive roles in the regulation of both systemic metabolism and reproduction, the integrated parts of which are worth further investigations.

In summary, our findings suggested that p62 deficiency in the pituitary led to female infertility via impaired LH, illustrating the GnRH-p62-OXPHOS (Ndufa2)-ATP-LH pathway in gonadotropic cells and highlighting the role of pituitary p62 in the female reproductive system. A balanced p62 level is required for basic activities and is essential in the crosstalk between metabolic and reproductive endocrine functions. In this way, our study extended the knowledge of p62 and provided a theoretical basis for further mechanical and therapeutic investigations into infertility.

## Supplementary information

Supplementary Information

## Data Availability

The datasets used and/or analyzed during the current study are available from the corresponding author on reasonable request.

## References

[1] Mascarenhas MN, Flaxman SR, Boerma T, Vanderpoel S, Stevens GA (2012). National, regional, and global trends in infertility prevalence since 1990: a systematic analysis of 277 health surveys. PLoS Med..

[2] Mikhael S, Punjala-Patel A, Gavrilova-Jordan L (2019). Hypothalamic-pituitary-ovarian axis disorders impacting female fertility. Biomedicines.

[3] World Health Organization. *International Classification of Diseases 11th Revision (ICD-11). Executive Board: EB143/13* (WHO, 2018).

[4] Weiss RV, Clapauch R (2014). Female infertility of endocrine origin. Arq. Bras. Endocrinol. Metabol..

[5] National Collaborating Centre for Women’s and Children’s Health. *Fertility: Assessment and Treatment for People with Fertility Problems. NICE Clinical Guidelines**No. 156* (National Collaborating Centre for Women’s and Children’s Health, 2013).

[6] Topaloglu AK, Kotan LD (2016). Genetics of hypogonadotropic hypogonadism. Endocr. Dev..

[7] Christensen A (2012). Hormonal regulation of female reproduction. Horm. Metab. Res..

[8] Wu S (2014). Obesity-induced infertility and hyperandrogenism are corrected by deletion of the insulin receptor in the ovarian theca cell. Diabetes.

[9] Xing C, Li C, He B (2020). Insulin sensitizers for improving the endocrine and metabolic profile in overweight women with PCOS. J. Clin. Endocrinol. Metab..

[10] Brothers KJ (2010). Rescue of obesity-induced infertility in female mice due to a pituitary-specific knockout of the insulin receptor. Cell. Metab..

[11] Manaserh IH (2019). Ablating astrocyte insulin receptors leads to delayed puberty and hypogonadism in mice. PLoS Biol..

[12] Rodriguez A (2006). Mature-onset obesity and insulin resistance in mice deficient in the signaling adapter p62. Cell Metab..

[13] Muller TD (2013). p62 links beta-adrenergic input to mitochondrial function and thermogenesis. J. Clin. Invest..

[14] Harada H (2013). Deficiency of p62/Sequestosome 1 causes hyperphagia due to leptin resistance in the brain. J. Neurosci..

[15] Duran A (2004). The atypical PKC-interacting protein p62 is an important mediator of RANK-activated osteoclastogenesis. Dev. Cell..

[16] Gieske MC (2008). Pituitary gonadotroph estrogen receptor-alpha is necessary for fertility in females. Endocrinology.

[17] Perez-Millan MI, Zeidler MG, Saunders TL, Camper SA, Davis SW (2013). Efficient, specific, developmentally appropriate cre-mediated recombination in anterior pituitary gonadotropes and thyrotropes. Genesis.

[18] Cushman LJ (2000). Cre-mediated recombination in the pituitary gland. Genesis.

[19] Goldman JM, Murr AS, Cooper RL (2007). The rodent estrous cycle: characterization of vaginal cytology and its utility in toxicological studies. Birth. Defects Res. B. Dev. Reprod. Toxicol..

[20] Cora MC, Kooistra L, Travlos G (2015). Vaginal cytology of the laboratory rat and mouse: review and criteria for the staging of the estrous cycle using stained vaginal smears. Toxicol. Pathol..

[21] Myers M, Britt KL, Wreford NG, Ebling FJ, Kerr JB (2004). Methods for quantifying follicular numbers within the mouse ovary. Reproduction.

[22] Ahmed K (2017). Loss of microRNA-7a2 induces hypogonadotropic hypogonadism and infertility. J. Clin. Invest..

[23] Fortune JE, Yang MY, Muruvi W (2010). The earliest stages of follicular development: follicle formation and activation. Soc. Reprod. Fertil. Suppl..

[24] Marieb, E. & Hoehn, K. in *Anatomy & physiology* 9th edn, Vol. 5 (eds. Beauparlant, S. & Puttkamer, G.) Ch. 27 (Pearson Education, 2013).

[25] Caldwell AS (2014). Characterization of reproductive, metabolic, and endocrine features of polycystic ovary syndrome in female hyperandrogenic mouse models. Endocrinology.

[26] van Houten EL, Visser JA (2014). Mouse models to study polycystic ovary syndrome: a possible link between metabolism and ovarian function?. Reprod. Biol..

[27] Maurya VK (2014). Expression and activity of Rac1 is negatively affected in the dehydroepiandrosterone induced polycystic ovary of mouse. J. Ovarian. Res..

[28] Wang H (2016). NRF2 activation by antioxidant antidiabetic agents accelerates tumor metastasis. Sci. Transl. Med..

[29] Huang da W, Sherman BT, Lempicki RA (2009). Systematic and integrative analysis of large gene lists using DAVID bioinformatics resources. Nat. Protoc..

[30] Monninkhof EM, Peeters PH, Schuit AJ (2007). Design of the sex hormones and physical exercise (SHAPE) study. BMC Public. Health.

[31] Turgeon JL, Kimura Y, Waring DW, Mellon PL (1996). Steroid and pulsatile gonadotropin-releasing hormone (GnRH) regulation of luteinizing hormone and GnRH receptor in a novel gonadotrope cell line. Mol. Endocrinol..

[32] Wu FC, Butler GE, Kelnar CJ, Sellar RE (1990). Patterns of pulsatile luteinizing hormone secretion before and during the onset of puberty in boys: a study using an immunoradiometric assay. J. Clin. Endocrinol. Metab..

[33] Yen SS, VandenBerg G, Rebar R, Ehara Y (1972). Variation of pituitary responsiveness to synthetic LRF during different phases of the menstrual cycle. J. Clin. Endocrinol. Metab..

[34] Lehman MN, Coolen LM, Goodman RL (2010). Minireview: kisspeptin/neurokinin B/dynorphin (KNDy) cells of the arcuate nucleus: a central node in the control of gonadotropin-releasing hormone secretion. Endocrinology.

[35] Zemkova H, Balik A, Jiang Y, Kretschmannova K, Stojilkovic SS (2006). Roles of purinergic P2X receptors as pacemaking channels and modulators of calcium-mobilizing pathway in pituitary gonadotrophs. Mol. Endocrinol..

[36] Stojilkovic SS, Bjelobaba I, Zemkova H (2017). Ion channels of pituitary gonadotrophs and their roles in signaling and secretion. Front. Endocrinol. (Lausanne).

[37] Thong EP, Codner E, Laven JSE, Teede H (2020). Diabetes: a metabolic and reproductive disorder in women. Lancet Diabetes Endocrinol..

[38] Broughton DE, Moley KH (2017). Obesity and female infertility: potential mediators of obesity’s impact. Fertil. Steril..

[39] Azziz R (2018). Polycystic ovary syndrome. Obstet. Gynecol..

[40] Escobar-Morreale HF (2018). Polycystic ovary syndrome: definition, aetiology, diagnosis and treatment. Nat. Rev. Endocrinol..

[41] Chen YH (2013). miRNA-93 inhibits GLUT4 and is overexpressed in adipose tissue of polycystic ovary syndrome patients and women with insulin resistance. Diabetes.

[42] Liu WJ (2016). p62 links the autophagy pathway and the ubiqutin-proteasome system upon ubiquitinated protein degradation. Cell. Mol. Biol. Lett..

[43] Moscat J, Karin M, Diaz-Meco MT (2016). p62 in cancer: signaling adaptor beyond autophagy. Cell.

[44] Zhu Y (2019). Autophagy in male reproduction. Syst. Biol. Reprod. Med..

[45] Gao H, Khawar MB, Li W (2019). Autophagy in reproduction. Adv. Exp. Med. Biol..

[46] Delcour C (2019). ATG7 and ATG9A loss-of-function variants trigger autophagy impairment and ovarian failure. Genet. Med..

[47] Henry, H. L. & Norman, A. W. *Encyclopedia of Hormones,* Vol.1 (Academic Press, 2003).

[48] Messager S (2005). Kisspeptin directly stimulates gonadotropin-releasing hormone release via G protein-coupled receptor 54. Proc. Natl Acad. Sci. USA.

[49] Kaniuga Z (1963). The transformation of mitochondrial Nadh dehydrogenase into Nadh: cytochrome C oxidoreductase. Biochim. Biophys. Acta.

[50] Amiott EA, Jaehning JA (2006). Mitochondrial transcription is regulated via an ATP “sensing” mechanism that couples RNA abundance to respiration. Mol. Cell.

[51] Jitrapakdee S, Wutthisathapornchai A, Wallace JC, MacDonald MJ (2010). Regulation of insulin secretion: role of mitochondrial signalling. Diabetologia.

[52] Naor Z (2009). Signaling by G-protein-coupled receptor (GPCR): studies on the GnRH receptor. Front. Neuroendocrinol..

[53] Chen ZP (1995). Evidence for a role of pituitary ATP receptors in the regulation of pituitary function. Proc. Natl Acad. Sci. USA.

[54] Chen ZP, Levy A, McArdle CA, Lightman SL (1994). Pituitary ATP receptors: characterization and functional localization to gonadotropes. Endocrinology.

[55] Huckle WR, Conn PM (1988). Molecular mechanism of gonadotropin releasing hormone action. II. The effector system. Endocr. Rev..

[56] Azziz R (2017). PCOS: Animal models for PCOS—not the real thing. Nat. Rev. Endocrinol..

