# Supplementary material for: Pituitary P62 deficiency leads to female infertility by impairing luteinizing hormone production
Source: Exp Mol Med. 2021 Aug 27;53(8):1238–49. doi: 10.1038/s12276-021-00661-4 (PMC8417229; doi:10.1038/s12276-021-00661-4)
Supplement: Supplementary file 1 — Supplementary Information [file 12276_2021_661_MOESM1_ESM.docx]

Supplemental Figure and Legends

**
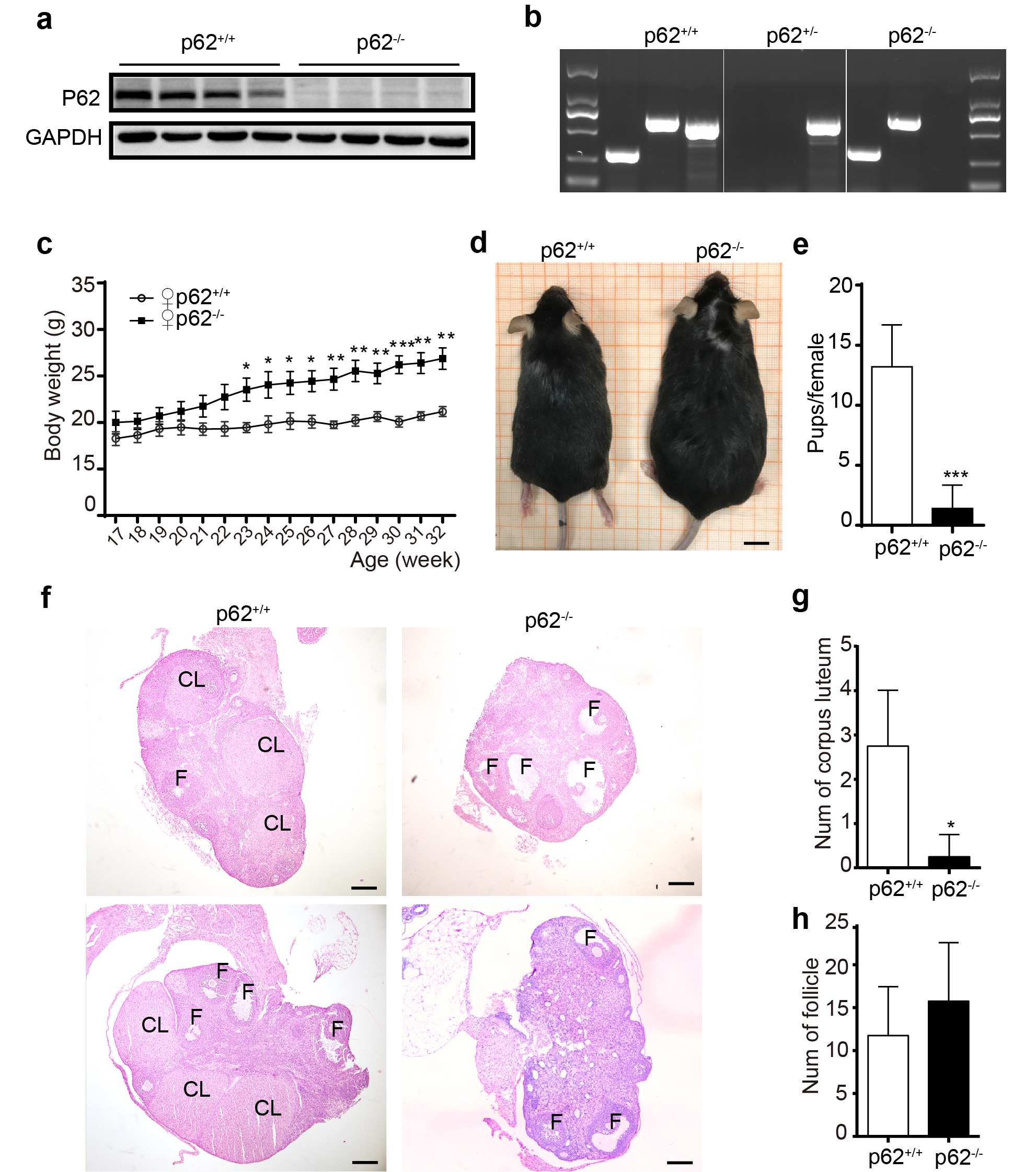
**

Supplementary Figure 1 Adult-onset obesity in p62^-/-^ mice is accompanied by reproductive dysfunction. **a, b** The target protein expression identification and genotyping for systemic p62 knockout female mice by WB and PCR. **c** Body weight growth curve of obesity in p62^-/-^ mice compared to p62^+/+^ mice from young age (8-week-old) to adult (8-month-old), n=5. **d** General morphology in 8-month-old adult p62^-/-^ mice (right) compared to p62^+/+^ mice (left). Scale bar: 1 cm. **e** Accumulative pups counting per female p62^+/+^ or p62^-/-^ mice hybridized with same aged male p62^+/+^ mice, during 22-week to 32-week-old, n=5. **f** Representative ovarian morphology of mice from each group detected by H&E staining. CL, corpus luteum; F, follicle; scale bar, 200 μm. **g, h** The numbers of corpus luteum and follicle counted from the H.E staining slices of ovaries. n=4. Data are shown as the mean ± SEM **(c)** and mean ± SD **(e, g, h).** Student’s *t* test. **P* ≤ 0.05; ***P* ≤ 0.01, ****P* ≤ 0.001.

**
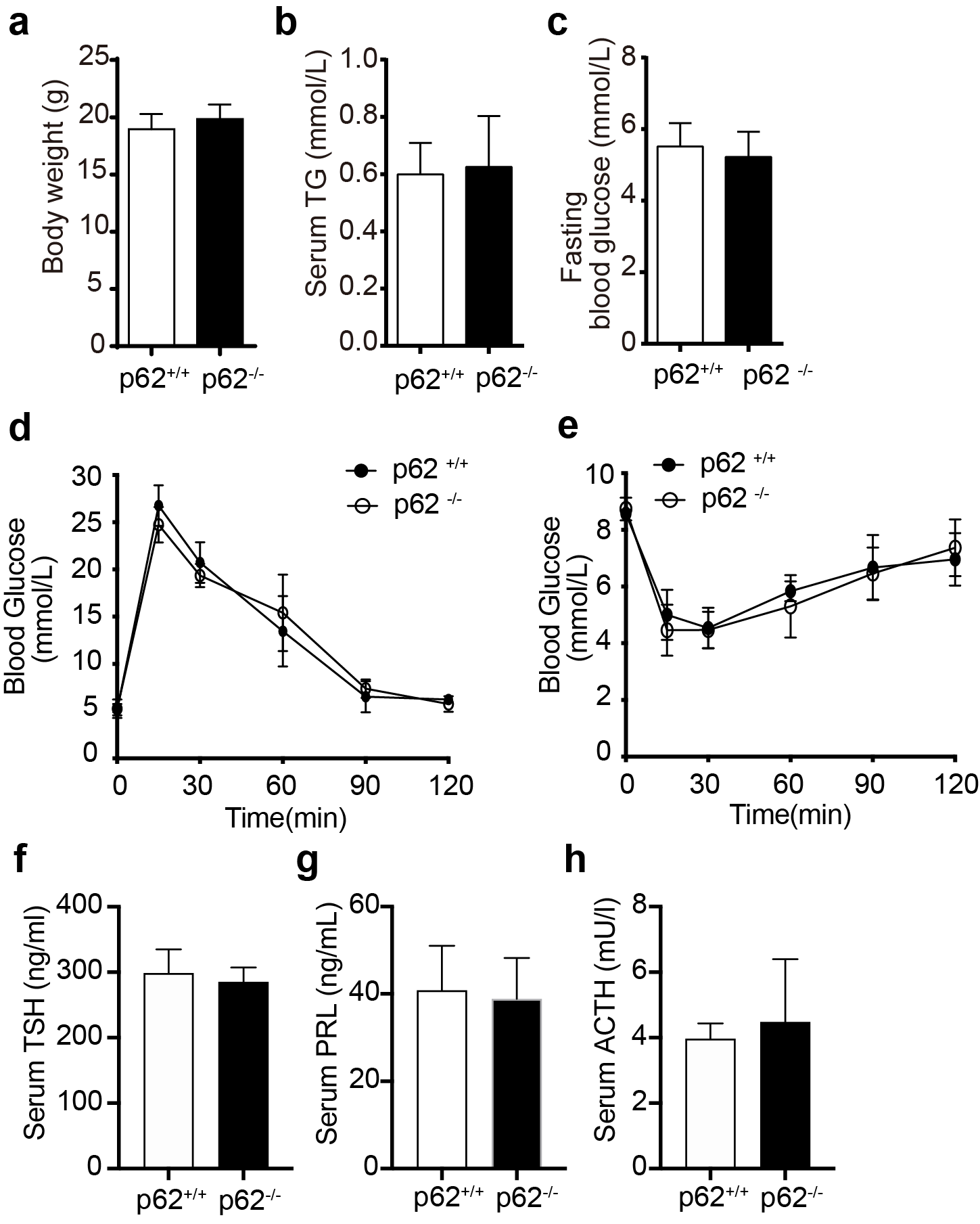
**

Supplementary Figure 2 No abnormalities of systemic metabolism and other pituitary hormones were observed in young nonobese p62^-/-^ mice. **a-c** Body weight, TG and fasting blood glucose between 8-week-old young female mice, n=4-5. **d, e** Blood glucose levels in Glucose tolerance test (GTT) and insulin tolerance test (ITT). **f-h** Serum TSH, PRL and ACTH levels in p62^-/-^ and control female mice, n=6-8. Data are shown as the mean ± SD, Student’s *t* test.

**
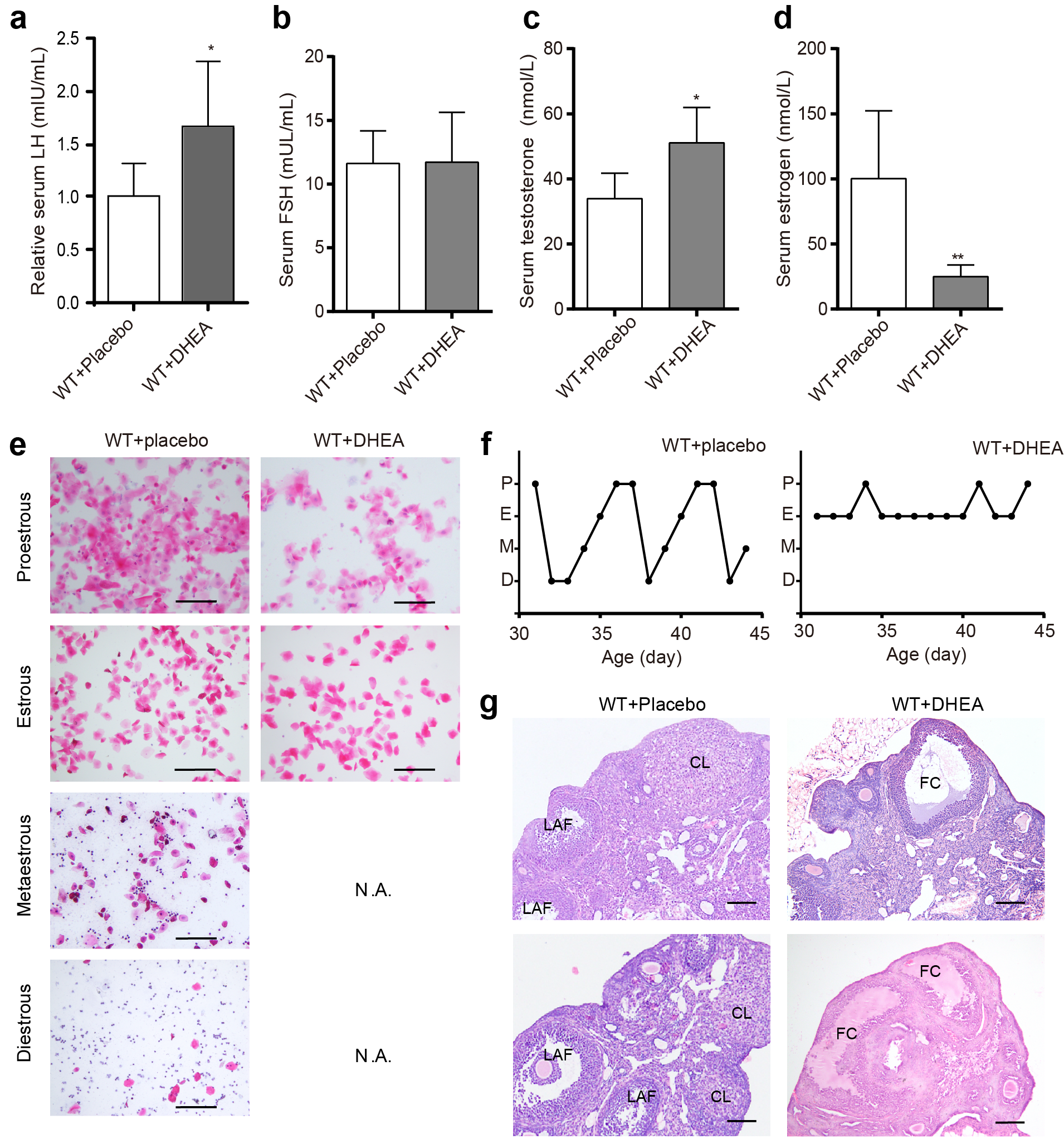
**Supplementary Figure 3 PCOS mice models display abnormal increased p62 and LH expression. **a-d** Relative serum LH, FSH, testosterone (T), and E2 levels in DHEA-induced-PCOS model (WT+DHEA) and control mice (WT+Placebo), n=5-7. **e-g** Representative vaginal cytology plot, corresponding estrus cycle and ovarian morphology of each group. CL, corpus luteum; LAF, large antral follicle; FC, follicular cyst. Data are shown as the mean ± SD. Student’s *t* test. **P* ≤ 0.05; ***P* ≤ 0.01.

**
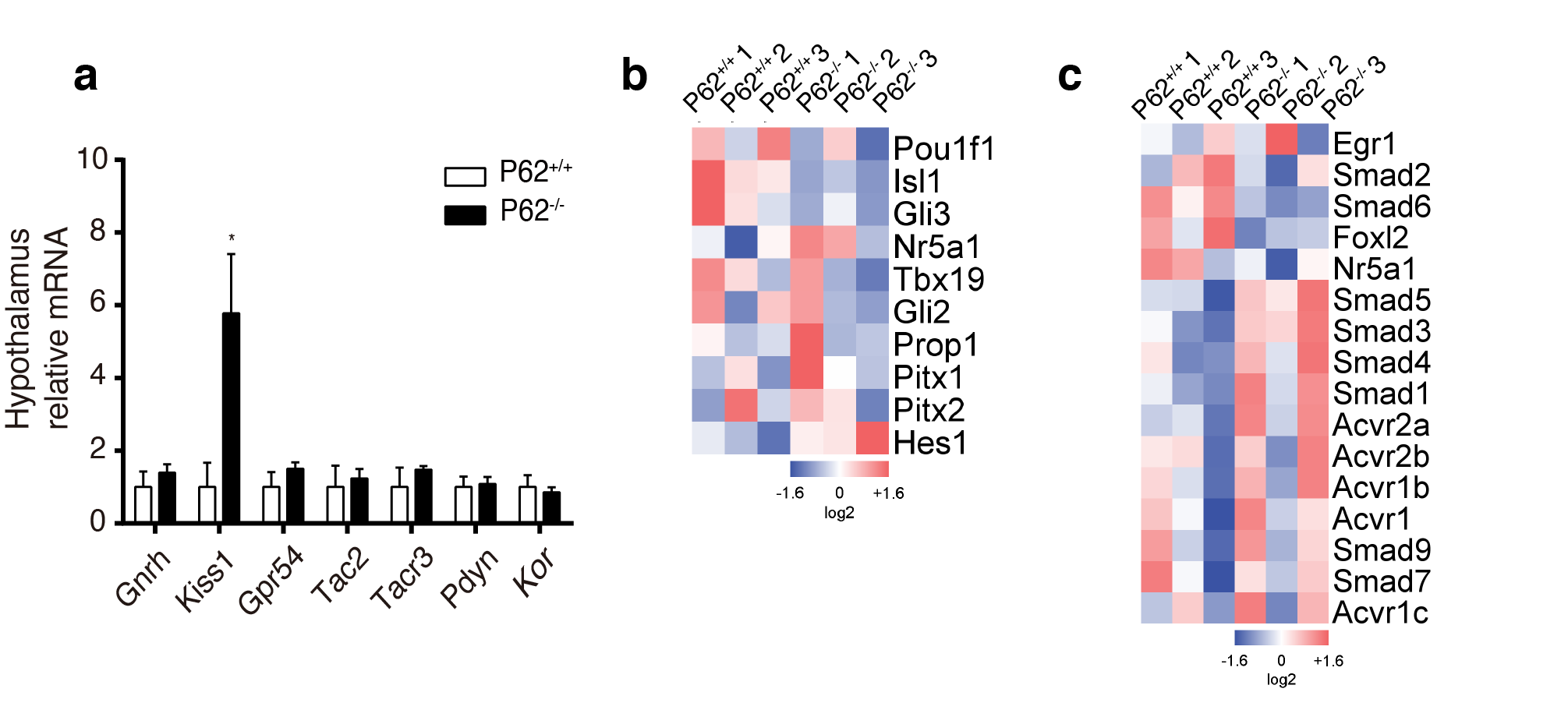
**

Supplementary Figure 4 The key factors in hypothalamus and pituitary. a RT-PCR detection in hypothalamus of relative expression of hypothalamic genes involved in GnRH-neuronal functions in each young mouse group, n=4-5. **b, c** RNA-seq detection of expression of early pituitary function and development in each young mouse group, shown as a heat map, n=3. Data are shown as the mean ± SEM. Student’s *t* test. **P* ≤ 0.05


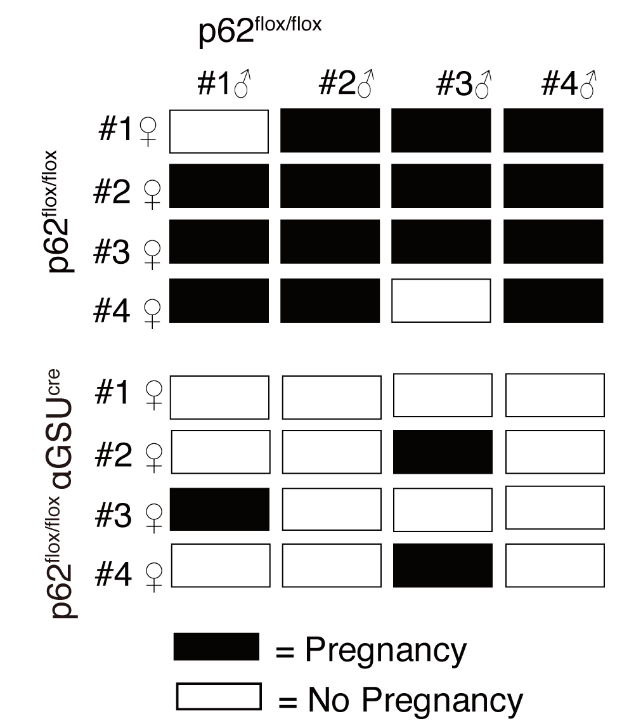


**Supplementary Figure 5** Infertility phenotype of pituitary-specific p62 knockout mice. Representative breeding experiment using young (8-weeks-old) pituitary-specific p62 knockout and control female mice (four p62^flox/flox^ αGSU^cre^ and four p62^flox/flox^), as well as adult male control mice (p62^flox/flox^) for 4 rounds, n=4. In the horizontal row, p62^flox/flox^ ♂#1-4 represents the control male mice that paired separately with female mice. In vertical column, p62^flox/flox^ #1-4♀ represents four control female mice, p62^flox/flox^ αGSU^cre^#1-4♀ represents four pituitary-specific p62 knockout female mice. Each bar represents one of the pairings: black bar means the successful pregnancy of female mice, and the blank bar means non-pregnancy.

Representative breeding study illustration: Four pituitary-specific p62 knockout female mice (p62^flox/flox^ αGSU^cre^) and four control female mice (p62^flox/flox^) were paired with adult control male mice (p62^flox/flox^) respectively (one female and one male mouse were paired in one cage) for 1 week, then the pairs were separated, and female mice returned to their own cages for 3 weeks to allow for birth of pups. For example, p62^flox/flox^ αGSU^cre^ ♀#1 were paired with p62^flox/flox^ ♂#1 for 1 week in one cage, then separated p62^flox/flox^ αGSU^cre^ ♀#1 to an empty cage for 3 weeks to allow for birth of pups. After one week of rest, p62^flox/flox^ αGSU^cre^ ♀#1 was paired with p62^flox/flox^ ♂#2 in this way, repeating this process until the female mice completed the pairing and breeding for 4 rounds.

**
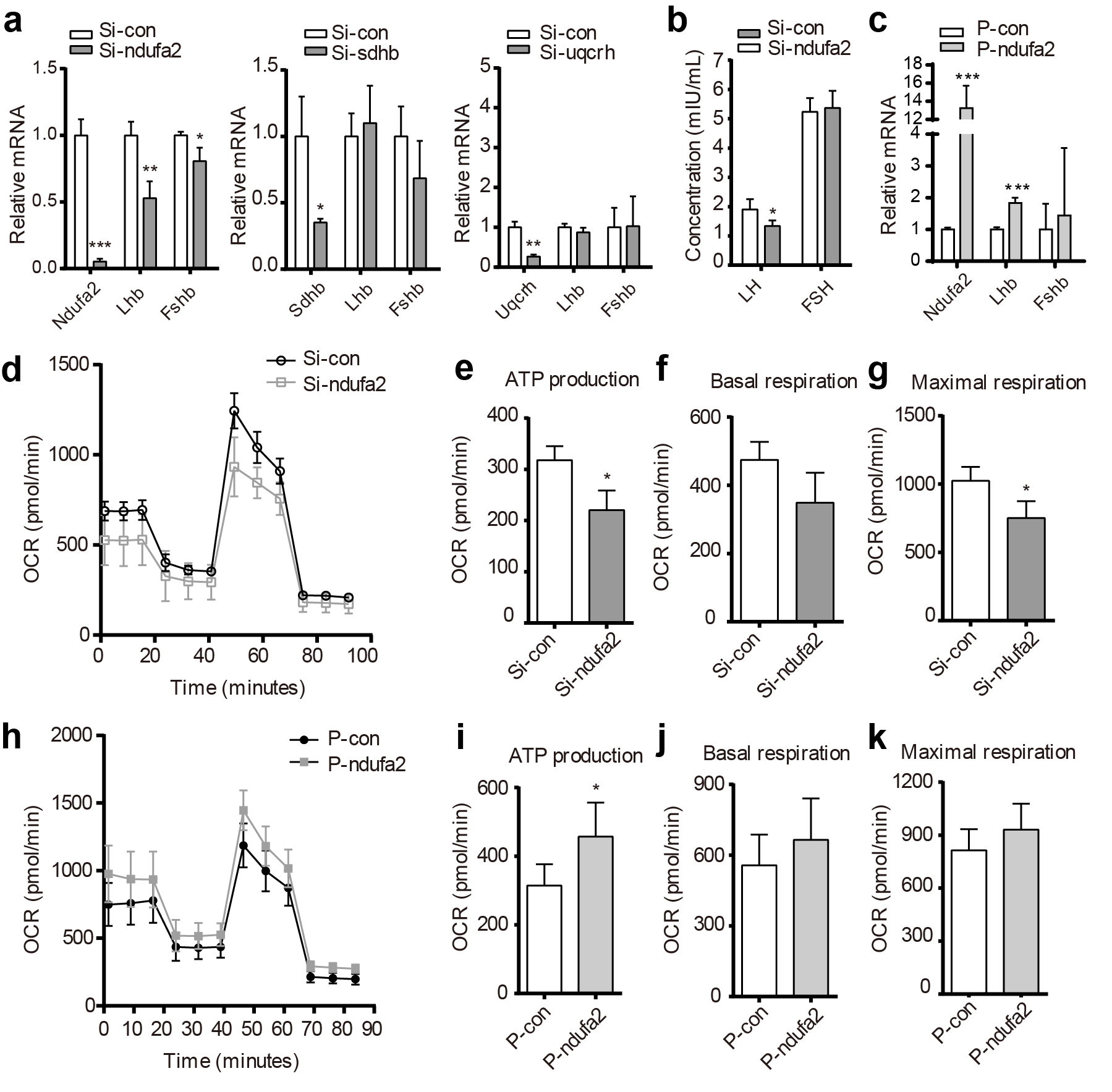
**Supplementary Figure 6 Ndufa2 positively modulates mitochondrial respiration and LH production. **a** mRNA detection of Ndufa2, Lhb, and Fshb in LβT2 cells transfected with Ndufa2, Sdhb or Uqcrh siRNA/plasmid for 48 h, n=3-4. **b** Supernatant measurement of LH and FSH in LβT2 cells transfected with Ndufa2 siRNA, n=3. **c** LβT2 cells transfected with Ndufa2 plasmid for 48 h, n=4. **d-k** Seahorse assay analysis for OCR, ATP production, basal and maximal respiration in LβT2 cells transfected with Ndufa2 siRNA **(d-g)** or plasmid **(h-k)**, n=5. Data are shown as the mean ± SD. Student’s *t* test. **P* ≤ 0.05; ***P* ≤ 0.01; ****P* ≤ 0.001.

**Supplementary Tables**

**Supplementary Table 1.** The key factors expression of early pituitary function and pituitary development in p62^-/-^ and p62^+/+^ mice.

| Early pituitary development | | | | | |
| --- | --- | --- | --- | --- | --- |
| Track_id | Gene_Name | Gene_Type | log2FC | Fold_Change | p_value |
| ENSMUSG00000048402.14 | Gli2 | protein_coding | 0.094704 | 1.067846 | 0.731064 |
| ENSMUSG00000021318.15 | Gli3 | protein_coding | 0.262053 | 1.199184 | 0.129109 |
| ENSMUSG00000026751.14 | Nr5a1 | protein_coding | -0.22453 | 0.855876 | 0.260401 |
| ENSMUSG00000021506.7 | Pitx1 | protein_coding | -0.27481 | 0.826558 | 0.355536 |
| ENSMUSG00000028023.16 | Pitx2 | protein_coding | 0.016662 | 1.011616 | 0.932748 |
| ENSMUSG00000042258.13 | Isl1 | protein_coding | 0.276845 | 1.211542 | 0.024564 |
| ENSMUSG00000026572.11 | Tbx19 | protein_coding | 0.325823 | 1.25338 | 0.458524 |
| ENSMUSG00000004842.18 | Pou1f1 | protein_coding | 0.397808 | 1.317505 | 0.217595 |
| ENSMUSG00000044542.3 | Prop1 | protein_coding | -0.12483 | 0.917114 | 0.64804 |
| ENSMUSG00000022528.7 | Hes1 | protein_coding | -0.37439 | 0.77143 | 0.065098 |
| Early pituitary function | | | | | |
| Track_id | Gene_Name | Gene_Type | log2FC | Fold_Change | p_value |
| ENSMUSG00000026836.15 | Acvr1 | protein_coding | 0.173014 | 1.127411 | 0.411677 |
| ENSMUSG00000000532.10 | Acvr1b | protein_coding | 0.325679 | 1.253254 | 0.398564 |
| ENSMUSG00000026834.13 | Acvr1c | protein_coding | 0.249746 | 1.188998 | 0.582919 |
| ENSMUSG00000052155.5 | Acvr2a | protein_coding | 0.768786 | 1.703836 | 0.113209 |
| ENSMUSG00000061393.14 | Acvr2b | protein_coding | 0.247982 | 1.187545 | 0.649053 |
| ENSMUSG00000038418.7 | Egr1 | protein_coding | -0.19728 | 0.872195 | 0.81876 |
| ENSMUSG00000050397.4 | Foxl2 | protein_coding | -0.46949 | 0.722221 | 0.05267 |
| ENSMUSG00000031681.16 | Smad1 | protein_coding | 0.429897 | 1.347137 | 0.078194 |
| ENSMUSG00000024563.15 | Smad2 | protein_coding | -0.20594 | 0.866973 | 0.310634 |
| ENSMUSG00000032402.12 | Smad3 | protein_coding | 0.611428 | 1.527771 | 0.033764 |
| ENSMUSG00000024515.13 | Smad4 | protein_coding | 0.413647 | 1.332049 | 0.126607 |
| ENSMUSG00000021540.16 | Smad5 | protein_coding | 0.806448 | 1.7489 | 0.073963 |
| ENSMUSG00000036867.7 | Smad6 | protein_coding | -0.46787 | 0.723029 | 0.009777 |
| ENSMUSG00000025880.11 | Smad7 | protein_coding | 0.055565 | 1.039266 | 0.754911 |
| ENSMUSG00000027796.2 | Smad9 | protein_coding | 0.134444 | 1.09767 | 0.54863 |
| ENSMUSG00000026751.14 | Nr5a1 | protein_coding | -0.22453 | 0.855876 | 0.260401 |

**Supplementary Table 2.** The OXPHOS markers expression in p62^-/-^ and p62^+/+^ mice pituitary tissue.

| Track_id | Gene_Name | Gene_Type | log2FC | Fold_Change | p_value |
| --- | --- | --- | --- | --- | --- |
| NADH dehydrogenase (Complex I) | | | |  |  |
| ENSMUSG00000022820.3 | Ndufb4 | protein_coding | -1.18183 | 0.440793 | 0.016145 |
| ENSMUSG00000016427.7 | Ndufa1 | protein_coding | -1.11114 | 0.462929 | 0.029807 |
| ENSMUSG00000014294.4 | Ndufa2 | protein_coding | -1.14774 | 0.451331 | 0.03089 |
| ENSMUSG00000035674.13 | Ndufa3 | protein_coding | -1.05085 | 0.482683 | 0.017082 |
| ENSMUSG00000029632.7 | Ndufa4 | protein_coding | -1.1179 | 0.460765 | 0.03571 |
| ENSMUSG00000041881.12 | Ndufa7 | protein_coding | -1.12375 | 0.4589 | 0.021625 |
| ENSMUSG00000036199.9 | Ndufa13 | protein_coding | -1.12583 | 0.45824 | 0.019636 |
| ENSMUSG00000031059.9 | Ndufb11 | protein_coding | -1.11358 | 0.462145 | 0.028491 |
| Succinate dehydrogenase/ Fumarate reductase (Complex II) | | | | | |
| ENSMUSG00000009863.14 | Sdhb | protein_coding | -0.6668 | 0.629904 | 0.047928 |
| Cytochrome C reductase (Complex III) | | | |  |  |
| ENSMUSG00000063882.12 | Uqcrh | protein_coding | -1.12164 | 0.459571 | 0.03317 |
| ENSMUSG00000044894.14 | Uqcrq | protein_coding | -1.10622 | 0.464509 | 0.036418 |
| ENSMUSG00000059534.8 | Uqcr10 | protein_coding | -1.04948 | 0.483141 | 0.018043 |
| ENSMUSG00000020163.12 | Uqcr11 | protein_coding | -1.21962 | 0.429396 | 0.017317 |
| Cytochrome C oxidase (Complex IV) | | | |  |  |
| ENSMUSG00000031818.12 | Cox4i1 | protein_coding | -0.73022 | 0.602811 | 0.031829 |
| ENSMUSG00000032330.7 | Cox7a2 | protein_coding | -1.41131 | 0.37597 | 0.035359 |
| ENSMUSG00000074218.3 | Cox7a1 | protein_coding | -1.40763 | 0.37693 | 0.008243 |
| ENSMUSG00000017778.14 | Cox7c | protein_coding | -1.20318 | 0.434318 | 0.03886 |
| ENSMUSG00000025488.9 | Cox8b | protein_coding | -1.17709 | 0.442242 | 0.043278 |
| ENSMUSG00000031231.4 | Cox7b | protein_coding | -1.08959 | 0.469895 | 0.043546 |
| ATP synthase/ F-type ATPase (Complex V) | | | | |  |
| ENSMUSG00000025781.14 | Atp5c1 | protein_coding | -0.80162 | 0.573704 | 0.017111 |
| ENSMUSG00000016252.7 | Atp5e | protein_coding | -1.16544 | 0.445829 | 0.043028 |
| ENSMUSG00000050856.16 | Atp5k | protein_coding | -1.25266 | 0.419675 | 0.016338 |
| ENSMUSG00000038690.15 | Atp5j2 | protein_coding | -1.22181 | 0.428743 | 0.025558 |
| ENSMUSG00000038717.8 | Atp5l | protein_coding | -1.06797 | 0.47699 | 0.021569 |

**Supplementary Table 3**. The genotyping primers for p62^-/-^ mice and p62^flox/flox^ αGSU^cre^ mice.

| p62^-/-^ genotyping | | |
| --- | --- | --- |
| Pair | Primer | Sequence |
| 1 | CSD-loxF | GAGATGGCGCAACGCAATTAATG |
|  | CSD-p62-R | ACACAACAAAACCTTGCTGGAGTCG |
| 2 | CSD-neoF | GGGATCTCATGCTGGAGTTCTTCG |
|  | CSD-p62-ttR | ACTAAGGCTCTACACTCAACCTGGC |
| 3 | CSD-p62-F | GGGCTACATACTAAGATCCTGCCCC |
|  | CSD-p62-ttR | ACTAAGGCTCTACACTCAACCTGGC |
| p62^flox/flox^ aGSU^cre^ genotyping | | |
| Pair | Primer | Sequence |
| 1 | CSD-p62-F | GGGCTACATACTAAGATCCTGCCCC |
|  | CSD-p62-ttR | ACTAAGGCTCTACACTCAACCTGGC |
| 2 | CSD-loxF | GAGATGGCGCAACGCAATTAATG |
|  | CSD-p62-R | ACACAACAAAACCTTGCTGGAGTCG |
| 3 | CSD-p62-F | GGGCTACATACTAAGATCCTGCCCC |
|  | CSD-p62-R | ACACAACAAAACCTTGCTGGAGTCG |
| 4 | oIMR3834 | ACATTGTTCCCCTCAGATCG |
|  | oIMR8990 | CAGGTTCTTGCGAACCTCAT |

F, forward primer; R, reverse primer.

**Supplementary Table 4.** Primers for RT-PCR.

| Target | Species | Forward primer | Reverse primer |
| --- | --- | --- | --- |
| p62 | Mouse | CCTTGCCCTACAGCTGAGTC | TTGTCTTCTGTGCCTGTGCT |
| Gapdh | Mouse | CTCCTCCTGTTCGACAGTCAGC | CCCAATACGACCAAATCCGTT |
| Lhcgr | Mouse | GATGCACAGTGGCACCTTC | GATGAGCGTCTGAATGGACTC |
| Fshr | Mouse | TGCCTGATGATGTTTTCCAG | GGCAGGGAATAGACCTTTGTC |
| StAR | Mouse | CCGGGTGGATGGGTCAA | CACCTCTCCCTGCTGGATGTA |
| P450scc | Mouse | CCATCAGATGCAGAGTTTCCAA | TGAGAAGAGTATCGACGCATCCT |
| Hsd17b2 | Mouse | TCACCAAGCCAGAGCAGATA | GTTAACCACGGCCCACAGT |
| Cyp19a1 | Mouse | CCACTCCTGCTGATCATGG | TCCCAGACAGTAGCCAGGAC |
| Pr | Mouse | CTCCGGGACCGAACAGAGT | ACAACAACCCTTTGGTAGCAG |
| Lhb | Mouse | CAAGAATGGAGAGGCTCCAG | ACTGGGCAGAACTCATTCTCTG |
| Fshb | Mouse | CAGGCAATCTTACGGTCTCG | GTGCGGGCTACTGCTACACT |
| Gnrhr | Mouse | GCCCCTTGCTGTACAAAGC | CCGTCTGCTAGGTAGATCATCC |
| Gnrh | Mouse | TCAGGGATCTGCGAGGAG | GGGCCAGTGCATCTACATC |
| Kiss1 | Mouse | ATGATCTCAATGGCTTCTTGG | CCAGGCATTAACGAGTTCCT |
| Gpr54 | Mouse | CCTTCACCGCACTCCTCTAC | CATACCAGCGGTCCACACTC |
| Tac2 | Mouse | CGTGACATGCACGACTTC | CCAACAGGAGGACCTTAC |
| Tac3r | Mouse | TACACCATCGTTGGAATTAC | ATGTCACCACCACAATAATC |
| Pdyn | Mouse | AGCTTGCCTCCTCGTGATG | GGCACTCCAGGGAGCAAAT |
| Kor | Mouse | TCCTTGGAGGCACCAAAGTCAGGG | TGGTGATGCGGCGGAGATTTCG |
| Ndufa1 | Mouse | ATGTGGTTCGAGATTCTCCCT | TGGTACTGAACACGAGCAACT |
| Ndufa2 | Mouse | TTGCGTGAGATTCGCGTTCA | ATTCGCGGATCAGAATGGGC |
| Ndufa3 | Mouse | ATGGCCGGGAGAATCTCTG | AGGGGCTAATCATGGGCATAAT |
| Ndufa4 | Mouse | TCCCAGCTTGATTCCTCTCTT | GGGTTGTTCTTTCTGTCCCAG |
| Sdhb | Mouse | ACCCCTTCTCTGTCTACCG | AATGCTCGCTTCTCCTTGTAG |
| Uqcrq | Mouse | CCTACAGCTTGTCGCCCTTT | GATCAGGTAGACCACTACAAACG |
| Uqcrh | Mouse | GCACTGTGAACAGCTGGAGA | TCTTCTGTCTGTGACCGGGA |
| Uqcr11 | Mouse | AAACTGGATTCCCACAGCCG | TGTAAGGCACCCAGTCCAGG |
| Cox4i1 | Mouse | ATTGGCAAGAGAGCCATTTCTAC | CACGCCGATCAGCGTAAGT |
| Cox7a1 | Mouse | GCTCTGGTCCGGTCTTTTAGC | GTACTGGGAGGTCATTGTCGG |
| Cox7a2 | Mouse | GCTGGCCCTTCGTCAGATT | GGCATCCCATTATCCTCCTGAA |
| Atp5c1 | Mouse | CCAGGAGACTGAAGTCCATCA | AGAACCTGTCCCATACACTCG |
| Atp5k | Mouse | GGTCTCTCCACTCATCAAGTTC | TCTCTCAATCCGTTTCAACTCA |

**Supplementary Table 5.** siRNA, shRNA and plasmid sequences.

| Target-vector | Species | Sense sequence/ transcript | Antisense sequence |
| --- | --- | --- | --- |
| p62 siRNA | Mouse | GACGAUGACUGGACACAUUTT | AAUGUGUCCAGUCAUCGUCTT |
| Ndufa2 siRNA | Mouse | ACAAUCUGAGUGCUGAUGATT | UCAUCAGCACUCAGAUUGUTT |
| Sdhb siRNA | Mouse | CCCUCUUCCACAUAUGUAUTT | AUACAUAUGUGGAAGAGGGTT |
| Uqcrh siRNA | Mouse | UGUGAUAAUCGCGUGUCUUTT | AAGACACGCGAUUAUCACATT |
| P62 shRNA | Mouse | GCTGAAACATGGACACTTTGG | - |
| Ndufa2 plasmid | Mouse | NM_010885.5 | - |
| Sdhb plasmid | Mouse | NM_001355515.1 | - |
| Uqcrh plasmid | Mouse | NM_025641.3 | - |
